# Multifaceted Roles of Cysteinyl Leukotrienes in Eliciting Eosinophil Granule Protein Secretion

**DOI:** 10.1155/2015/848762

**Published:** 2015-03-19

**Authors:** Renata Baptista-dos-Reis, Valdirene S. Muniz, Josiane S. Neves

**Affiliations:** Institute of Biomedical Sciences, Federal University of Rio de Janeiro, Centro de Ciências da Saúde (CCS), 373 Carlos Chagas Filho Avenue, Room F 14, 1st Floor, Ilha do Fundão, 21941-590 Rio de Janeiro, RJ, Brazil

## Abstract

Cysteinyl leukotrienes (cysLTs) are cell membrane-impermeant lipid mediators that play major roles in the pathogenesis of eosinophilic inflammation and are recognized to act via at least 2 receptors, namely, cysLT_1_ receptor (cysLT_1_R) and cysLT_2_ receptor (cysLT_2_R). Eosinophils, which are granulocytes classically associated with host defense against parasitic helminthes and allergic conditions, are distinguished from leukocytes by their dominant population of cytoplasmic crystalloid (also termed secretory, specific, or secondary) granules that contain robust stores of diverse preformed proteins. Human eosinophils are the main source of cysLTs and are recognized to express both cysLTs receptors (cysLTRs) on their surface, at the plasma membrane. More recently, we identified the expression of cysLTRs in eosinophil granule membranes and demonstrated that cysLTs, acting via their granule membrane-expressed receptors, elicit secretion from cell-free human eosinophil granules. Herein, we review the multifaceted roles of cysLTs in eliciting eosinophil granule protein secretion. We discuss the intracrine and autocrine/paracrine secretory responses evoked by cysLTs in eosinophils and in cell-free extracellular eosinophil crystalloid granules. We also discuss the importance of this finding in eosinophil immunobiology and speculate on its potential role(s) in eosinophilic diseases.

## 1. Introduction 

Cysteinyl leukotrienes (cysLTs) constitute an important class of potent proinflammatory mediators. These leukotrienes are synthesized from membrane-derived arachidonic acid via the 5-lipoxygenase (5-LO) pathway in concert with the 5-LO-activating protein (FLAP), forming LTA_4_, which is converted into LTC_4_ by the action of LTC_4_ synthase [1]. The production of LTC_4_ within cells may occur at perinuclear membranes or in cytoplasmic lipid bodies, which are cytoplasmic organelles rich in lipids. The formation of these bodies is highly regulated, and they have functions in eicosanoid production and storage of inflammatory proteins [2]. Intracellular LTC_4_ is actively transported extracellularly, where it is enzymatically sequentially converted to LTD_4_ and then to LTE_4_ [1]. CysLTs are cell membrane-impermeant and are recognized to mediate their actions by engaging at least two heptahelical G protein-coupled receptors (GPCRs), designated cysLT_1_ receptor (cysLT_1_R) and cysLT_2_ receptor (cysLT_2_R), which are expressed on the cell surface, at the plasma membrane [1, 3]. The rank orders of the affinities of cysLTs for human cysLT_1_R and cysLT_2_R, based on research in transfected cells, are LTD_4_ ≫ LTC_4_ = LTE_4_ and LTC_4_ = LTD_4_ > LTE_4_, respectively [4, 5]. However, various findings suggest the existence of another not yet cloned cysLT receptor (cysLTR), since numerous cysLTs' biological actions are not well explained by their affinities to the known cysLTRs [6–11]. For instance, experiments in animal models and human studies have revealed that LTE_4_, considered the weakest cysLTRs agonist, has unique characteristics that cannot be explained by current knowledge of cysLT_1_R and cysLT_2_R [9–11]. Moreover, an additional receptor sensitive to LTE_4_, the purinergic P2Y12 receptor (P2Y12R), has been identified by* in silico *and* in vivo* methods [12, 13]. In contrast, other studies have suggested that LTE_4_, as well as other cysLTs, does not activate intracellular signaling by acting through P2Y12R and that another LTE_4_-specific receptor has yet to be identified [14]. In fact, homo- and heterodimerization of cysLTRs and purinergic receptors have been widely suggested [15, 16]. Whether the 3 candidate cysLTRs function or interact as homo- or heterodimers is not known. More studies are still needed to better clarify this point.

Human eosinophils are major sources of cysLTs and express both cysLT_1_R and cysLT_2_R on their plasma membranes [1, 17]. CysLTs and their receptors have critical roles in allergic diseases and represent important therapeutic targets for the control of asthma and other pathophysiological conditions [15, 18]. Within eosinophils, synthesis of LTC_4_ (but not extracellularly formed LTD_4_ or LTE_4_) occurs at perinuclear membranes and in cytoplasmic lipid bodies [17]. Mature eosinophils contain a single population of secondary (or specific or crystalloid) granules that are ultrastructurally characterized as membrane-bound organelles containing a crystalloid core surrounded by a matrix. Based on diverse electron microscopy and subcellular fractionation studies, it is now recognized that human eosinophils synthesize and store cationic proteins, such as eosinophil cationic protein (ECP), eosinophil-derived neurotoxin (EDN), eosinophil peroxidase (EPO), eosinophil granule major basic protein 1 (MBP-1), enzymes, growth factors, chemokines (such as RANTES and eotaxin), and over 36 cytokines (including Th1 and Th2 cytokines) that are selectively secreted in response to a range of stimuli and agonists [19–26]. Mechanisms for differentially mobilizing these granule-stored proteins for their extracellular release may enable eosinophils to selectively and rapidly influence various immune, inflammatory, and other responses. The secretion of granule contents from intact eosinophils primarily occurs by a mechanism termed piecemeal degranulation (PMD). This is a process whereby granule contents are selectively mobilized into spherical and tubular vesicles that need to disengage from the granules, transit through the cytoplasm, and fuse with the plasma membrane to release their specific granule-derived protein cargo at the cell surface [21, 23, 27]. Another mechanism of human eosinophil “degranulation” is associated with cytolysis. Following lysis of an eosinophil, with loss of its plasma membrane integrity, intact, cell-free, membrane-bound granules are released and deposited extracellularly. Although PMD is considered to be the predominant mechanism underlying eosinophil degranulation and secretion, cytolysis has been recognized as a common mechanism for cell-free eosinophil granule release and deposition in tissues in eosinophilic diseases [28–31]. Compound exocytosis, whereby the entire granule contents are released extracellularly following fusion of the granules with the plasma membrane, occurs when eosinophils interact with large targets, such as helminthic parasites. However, this process is not usually observed* in vivo*.

Given that the selective release of cytokines can provide a mean for eosinophils to rapidly influence adjacent cells in normal or inflamed tissues, investigation of the mechanisms involved in the selective mobilization and vesicle-mediated secretion of specific cytokines, including IL-4 and other preformed cytokines, is extremely relevant. Several studies have provided new insights into the signal transduction processes that contribute to the selective mobilization and release of specific eosinophil granule-derived cytokines and chemokines [22, 32–36]. Several of these studies have identified the intracellular expression of cysLTRs and cysLT production as important inflammatory mediators eliciting the secretion of specific cytokines from eosinophils and from cell-free extracellular eosinophil granules [32–34, 36]. It appears increasingly likely that eicosanoids synthesized within cells, including eosinophils, may have other important intracrine roles in regulating cell functions, in addition to their more recognized autocrine/paracrine activities in inflammation.

## 2. CysLTs Are Intracrine Signals Regulating Eosinophils' IL-4 Secretion by Piecemeal Degranulation

**Figure 1:**
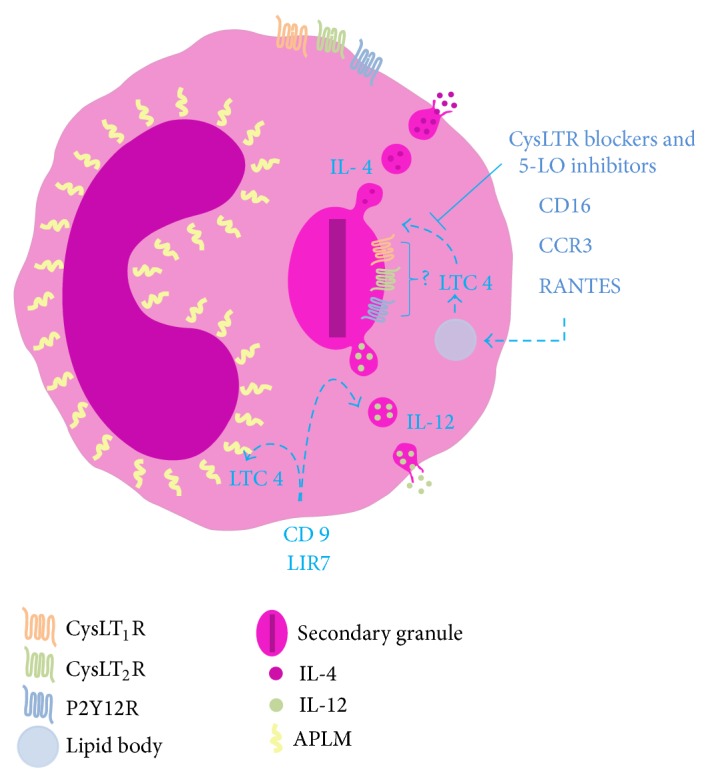
CysLTs are intracrine signals regulating eosinophils' IL-4 secretion by piecemeal degranulation.IL-4 release induced by IL-16, eotaxin, and RANTES is dependent on the intracrine action of lipid body-generated LTC_4_. Inhibitors of 5-lypoxigenase (5-LO) and/or cysteinyl leukotriene receptors (cysLTRs) blocked intracellular LTC_4_ production and consequently IL-4 release from eosinophils. The cross-linking of LIR7- or CD9-induced perinuclear-generated LTC_4_, however IL-12 secretion induced by LIR7 or CD9 is independent of 5-LO metabolites. CysLT_1_R = cysLT_1_ receptor; cysLT_2_R = cysLT_2_ receptor, P2Y12R = purinergic P2Y12 receptor, and APLM = arachidonyl phospholipids and lipoxygenase machinery.

In eosinophils, it is noteworthy that in addition to their recognized activities as autocrine/paracrine mediators, eicosanoids such as cysLTs are now also recognized to display intracrine effects. The cysLTs, LTC_4_, and their extracellular derivatives, LTD_4_ and LTE_4_, are recognized as paracrine mediators pertinent to asthma and allergic diseases based on their receptor-mediated capabilities to elicit bronchoconstriction, mucous hypersecretion, bronchial hyperresponsiveness, increased microvascular permeability, and additional eosinophil infiltration [1, 15, 18, 37]. Eosinophils are major sources of cysLTs [17] and are the principal LTC_4_ synthase-expressing cells in bronchial mucosa biopsy specimens from asthmatic subjects, as well as being recognized to express both cysLTRs [1, 17]. Thus, cysLTs are also important autocrine regulators of eosinophil function. Indeed, a series of reports showed that cysLTs have the ability to affect various eosinophil responses [35, 38–41]. For instance, in eosinophils derived from human cord blood progenitors* in vitro*, it was shown that LTC_4_, LTD_4_, and LTE_4_ induced dose- and time-dependent, vesicular transport-mediated release of preformed IL-4 [38]. Although controversy exists [39], cysLTs also appear to be able to induce the* in vitro* survival of human eosinophils by activation of cysLT_1_R [40, 41]. Additionally it was demonstrated that enhanced plasma membrane expression of activation-related CD69 on human eosinophils induced by platelet-activating factor (PAF) and IL-5 is dependent on endogenous eosinophil-derived 5-LO metabolites [35]. Consequently, much interest in understanding the regulation of eicosanoid formation in eosinophils has focused on the mechanisms that regulate eosinophil cysLT formation and release. Interestingly, it was noted that depending on the stimulus, the localized synthesis of LTC_4_ may occur at distinct intracellular sites within eosinophils (at the perinuclear membrane and/or in lipid bodies) and may control the role of this mediator as either an intracrine signal-transducing mediator that regulates PMD and cytokine secretion or an autocrine/paracrine element in eosinophilic inflammation [2, 32, 36]. In 2002, Bandeira-Melo and colleagues [32] evaluated whether cysLTs function as intracrine mediators involved in the stimulated release of IL-4 from eosinophils. The authors demonstrated that although eotaxin and RANTES each act via CCR3 to stimulate the secretion of both IL-4 and RANTES from eosinophils, only the release of IL-4 was dependent on the activation of 5-LO to form LTC_4_ within eosinophils' lipid bodies. Inhibitors of 5-LO blocked IL-16-, eotaxin-, and RANTES-induced IL-4 release, but exogenous LTC_4_, LTD_4_, and LTE_4_ did not elicit IL-4 release. Only after membrane permeabilization were cysLTs enabled to enter eosinophils and stimulate IL-4 but not RANTES release. LTC_4_- and LTD_4_-elicited IL-4 release was pertussis toxin inhibitable, but inhibitors of the two known GPCRs, cysLT_1_R and cysLT_2_R, did not block LTC_4_-elicited IL-4 release. LTC_4_ was more potent than LTD_4_ was and, at low concentrations, elicited IL-4 release from permeabilized eosinophils, whereas higher concentrations were inhibitory probably due to the high-dose inhibition characteristic of the GPCRs. For intact eosinophils, also as a consequence of high intracellular LTC_4_ levels, LTC_4_ export inhibitors blocked eotaxin-elicited IL-4 release. Thus, taken together, these data demonstrate that despite being well recognized as an autocrine/paracrine mediator, LTC_4_, via an intracellular cysLTR distinct from cysLT_1_R and cysLT_2_R, may also dynamically govern inflammatory responses as an intracrine mediator of eosinophils' PMD-mediated cytokine secretion (Figure 1).

Interestingly, in a different study, Tedla and colleagues showed that the cross-linking of leukocyte immunoglobulin-like receptor 7 (LIR7) and CD9 with immobilized antibodies induced LTC_4_ generation at the nuclear envelope and the release of IL-12, but not IL-4, by vesicular transport [36]. Whereas the IL-4 release induced by IL-16 and CCR3-activating chemokines is dependent on the intracrine action of lipid body-generated LTC_4_ [32], the IL-12 release induced by the cross-linking of LIR7 does not appear to be regulated by 5-LO metabolites [36, 42]. Pretreatment with two mechanistically distinct inhibitors of 5-LO (AA861 and MK886) blocked IL-16-, eotaxin-, and RANTES-induced LTC_4_ production and IL-4 release from eosinophils [32]. In contrast, pretreatment of eosinophils with either AA861 or MK886 did not inhibit the selective release of IL-12 induced by the cross-linking of CD9 or LIR7, indicating that 5-LO does not participate in CD9- or LIR7-driven selective IL-12 release [36, 42]. Moreover, stimulation of permeabilized eosinophils with LTC_4_ did not elicit IL-12 release [36]. Overall, intracellular LTC_4_ formed in lipid bodies appears to function as an intracrine, and not an extracellular autocrine/paracrine, mediator to regulate the differential secretion of IL-4 induced by IL-16, eotaxin, or RANTES [32]. Meanwhile, the intracellular 5-LO-derived LTC_4_ formed at the perinuclear membrane appears not to control the selective IL-12 release induced by the cross-linking of CD9 or LIR7 and may function as an autocrine/paracrine mediator of inflammation [36]. These studies suggest that the capacity of eosinophils to synthesize LTC_4_ in lipid bodies may relate less to paracrine mediator formation and more to intracrine signal-transducing activities pertinent to more local transcriptional or other cellular functions [43] (Figure 1).

Possible intracrine roles for LTC_4_ have also been described in other cell types, including vascular and mast cells; however, how LTC_4_ acts intracellularly remains to be defined [44–46]. Although eosinophils express the two known cysLTRs, cysLT_1_R and cysLT_2_R [17, 42], little is known about the intracellular distribution of these receptors in eosinophils. In addition to its conventional plasma membrane expression, cysLT_1_R has been immunolocalized to nuclei in colorectal adenocarcinoma cells [47], in a human mast cell line [48] and in vascular smooth muscle cells [46]. The functions of nuclear cysLT_1_R are poorly understood. For instance, in one interesting study, Nielsen and colleagues demonstrated that isolated intestinal cell nuclei express cysLT_1_R and respond to LTD_4_, triggering ERK1/2 signaling [47]. However, whether these nuclear-localized receptors are involved in the cell cycle (for survival or proliferation) is still unknown. In a different study, Eaton and colleagues showed that LPS upregulated the perinuclear expression of cysLT_1_R in vascular smooth muscle cells and that LTC_4_ stimulation predominantly enhanced nuclear calcium increase and gene transcription [46]. Whether or how exogenous LTC_4_ reaches these intracellular cysLT_1_Rs is still not defined. Recently, we defined the intracellular expression of cysLT-sensitive receptors in crystalloid granule membranes [34]. These findings might help in identifying novel mechanisms whereby cysLTs can serve as intracrine mediators.

## 3. Extracellular Eosinophil Granules Express Ligand-Binding Domains for CysLTRs on Their Membranes and Secrete ECP in Response to CysLTs

**Figure 2:**
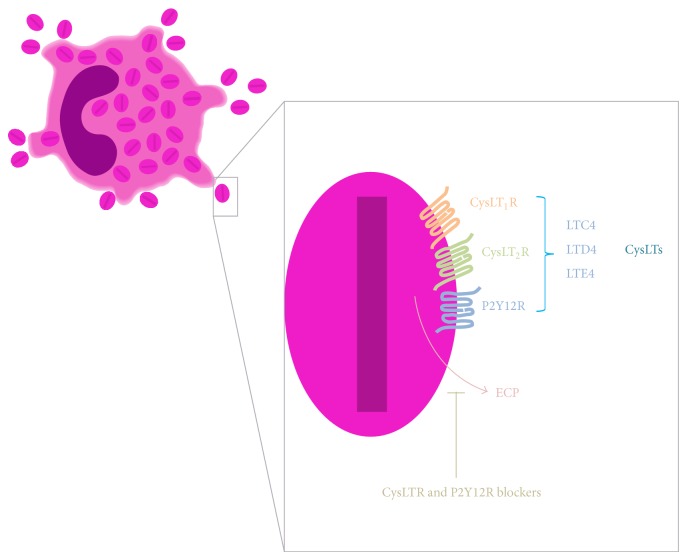
Stimulating cell-free eosinophil granules with the agonists LTC_4_, LTD_4_, and LTE_4_ elicited the secretion of eosinophil cationic protein (ECP) but not eosinophil-derived cytokines or chemokines from the granules. Cysteinyl leukotriene receptor (cysLTR) or P2Y12 receptor (P2Y12R) blockers inhibited ECP secretion after LTC_4_/LTD_4_/LTE_4_ stimulation of cell-free eosinophil granules.

Intracrine roles for cysLTs are described in the literature, but the mechanisms involved that can explain cysLTs' intracellular actions remain unknown [32, 44, 45]. A description of intracellular cysLTRs expression in human eosinophils was recently provided by our group [34]. In 2010, we reported, for the first time, that the receptors for cysLTs, cysLT_1_R and cysLT_2_R, and the purinergic P2Y12R are expressed on eosinophil granule membranes [34]. We showed that eosinophil granules express amino-terminal, ligand-binding domains for cysLT_1_R and cysLT_2_R and the P2Y12R on their membranes. We previously observed that certain cytokine and chemokine receptors are richly present on eosinophil granules [22, 33, 49]. These granules, upon extrusion from eosinophils, responded to a stimulating cytokine, interferon-*γ*, and a chemokine, eotaxin-1 (CCL11), via cognate granule membrane-expressed receptors to activate intragranular signaling pathways that elicit granule protein secretion [33, 50]. Stimulating cell-free eosinophil granules with the agonists LTC_4_, LTD_4_, and LTE_4_ elicited the secretion of ECP, but not eosinophil-derived cytokines or chemokines, from the granules (as detected by cytokine multiplex assays). Montelukast, a recognized inhibitor that principally inhibits cysLT_1_R, as well as the P2Y12R antagonist MRS 2395, inhibited eosinophil granule ECP secretion after LTC_4_/LTD_4_/LTE_4_ stimulation of cell-free eosinophil granules [34] (Figure 2). The capacity of a cysLT_1_R inhibitor or a P2Y12R antagonist, such as montelukast and MRS 2395, respectively, to similarly inhibit the secretion elicited by ligands (e.g., LTE_4_) not active for cysLT_1_R or not classically selective for the receptor (e.g., P2Y12R), suggests functional heterodimerization of cysLT_1_R and other receptors (e.g., functional heterodimerization between cysLT_1_R, cysLT_2_R, and P2Y12R) expressed on eosinophil granule membranes; whether this is the case remains to be ascertained. In addition, montelukast's potential off-target effects could not be discounted. Notably, the dose response to the three cysLTs varied. LTC_4_ and LTE_4_ elicited ECP secretion only at lower (subnanomolar) concentrations, which was fully consistent with the high-dose inhibition characteristic of the GPCRs. Intriguingly, LTD_4_ elicited ECP secretion at low and high, but not intermediate, concentrations. This dose response suggests the engagement of two receptors sensitive to LTD_4_, with the first responding to low LTD_4_ levels and then exhibiting higher dose inhibition and the second receptor putatively mediating secretion at higher concentrations of LTD_4_. As previously mentioned, oligomerization of leukotriene and purinergic receptors has been widely suggested [15, 16]. However, whether dimerization of receptors is involved in this response remains to be elucidated. These findings highlight the capacity of cysLTRs to stimulate cell-free granule secretory responses. Furthermore, for granules serving as intracellular organelles these data identify novel mechanisms whereby LTC_4_ and extracellularly generated LTD_4_ and LTE_4_ (if these mediators could be, by any chance, internalized by the cell) may serve as intracrine mediators of eosinophil granule-derived secretion. However, there is no evidence that the cysLT_1_R, cysLT_2_R, or P2Y12R expressed on granule membranes is involved in the intracrine actions of cysLTs described previously [32]. This phenomenon is not likely, considering that LTC_4_- and LTD_4_-elicited IL-4 release in permeabilized eosinophils is not blocked by inhibitors of cysLT_1_R and cysLT_2_R [32].

All of these findings are remarkable because they provide additional information about the capacity of eosinophils to contribute to modulating host and inflammatory responses after eosinophil cytolysis. Cytolytic release of intact eosinophil granules yields extracellular organelles fully capable of ligand-elicited active secretory responses, amplifying the differential secretory properties of eosinophils and likely contributing to the persistence and exacerbation of the inflammatory response.

## 4. Implications for Eosinophilic Diseases and Eosinophil Immunobiology: Questions for the Future

Intact membrane-bound eosinophil granules have long been recognized to be present extracellularly in tissues and secretions in many human eosinophil-enriched disorders (for review see [49]). The capacity of cell-free human eosinophil granules to act via receptor-mediated responses to cysLTs and to secrete granule-derived cationic proteins has indicated that cell-free eosinophil granules may be functionally significant in response to these lipid mediators [34], likely contributing to perpetuation of the inflammatory process in an affected organ. As intracrine mediators, the capacity of eicosanoids, such as LTC_4_, to be synthesized at 2 discrete sites, perinuclear membranes and lipid bodies, in eosinophils raises questions about the roles of these pools of lipids in the functioning of eosinophils in inflammation. As noted above, cysLTs are well-recognized autocrine/paracrine mediators that are pertinent to eosinophils, asthma, and allergic inflammation; however, how these eicosanoids transit from their intracellular sites of synthesis for their extracellular release, which is requisite for their autocrine/paracrine functions, remains uncertain. This uncertainty is especially true for LTC_4_ formed at perinuclear membranes that are not proximate to the cell surface. Furthermore, the logic of using phospholipids resident in perinuclear membranes as a source of substrate for forming secreted eicosanoid mediators remains unclear. Therapeutic agents in current use that inhibit 5-LO or cysLT_1_R may have activities broader than simply blocking the paracrine-mediator activities of cysLTs. Furthermore, with greater understanding of the intracellular sites of cysLT synthesis and cysLTR expression and of the functional consequences of such cysLTR-mediated cellular regulation for both intracellular and cell-free granules, newer therapeutic agents may be targeted to regulate specific activities of cysLTs pertinent to asthma and other allergic diseases.

Notably, studies demonstrating that isolated human eosinophil granules could exert extracellular functions as secretion-competent organelles after stimulation with ligands, including chemokines and cysLTs [33, 34], provide highly relevant observations in terms of eosinophil cell biology. Considering functional roles for these intracellular granule membrane-expressed receptors, with their ligand-binding domains displayed on the outer granule membranes, these findings not only support extracellular granule function but also suggest that the granule-expressed receptors potentially serve as intracrine mediators of eosinophil-derived granule secretion. For receptors such as the lipid-mediator receptors (cysLT_1_R, cysLT_2_R, and P2Y12R), which are activated by hydrophobic ligands that can be synthesized at the nuclear membrane or in lipid bodies, it is possible to predict roles in intracellular compartments, where they would be accessible to their ligands. For chemokine/cytokine receptors, it is also feasible that the ligands themselves are active intracellularly after their biosynthesis and/or based on specific cell uptake and internalization mechanisms.

Current investigations are only beginning to explore the functional biology and responses of eosinophil granules. Most notably, how specific granule-derived proteins are selectively mobilized for secretion from either isolated eosinophil granules or intracellular granules by PMD within intact eosinophils is yet to be ascertained. The molecular mechanisms that regulate eosinophil granule protein mobilization and secretion continue to be intriguing and requiring further delineation.
